# Long non-coding RNA THRIL inhibits miRNA-24-3p to upregulate neuropilin-1 to aggravate cerebral ischemia-reperfusion injury through regulating the nuclear factor κB p65 signaling

**DOI:** 10.18632/aging.202762

**Published:** 2021-03-06

**Authors:** Feng Kuai, Liang Zhou, Jianping Zhou, Xuemei Sun, Wanli Dong

**Affiliations:** 1 Department of Neurology, The First Affiliated Hospital of Soochow University, Suzhou 215006, China; 2 Department of Geriatrics, The First People’s Hospital of Yancheng, The Forth Affiliated Hospital of Nantong University, Yancheng 224001, China; 3 Department of orthopedic, The People's Hospital of Lianshui, Huai'an 223001, China

**Keywords:** cerebral ischemia-reperfusion injury, TNF and HNRNPL related immunoregulatory lncRNA, miR-24-3p, neuropilin-1, nuclear factor κB p65 signaling

## Abstract

Purpose: The aim of this study was to investigate the role of the tumor necrosis factor and HNRNPL related immunoregulatory long non-coding RNA (THRIL) in cerebral ischemia-reperfusion injury.

Methods: A rat middle cerebral artery occlusion/ischemia-reperfusion (MCAO/IR) model and an oxygen glucose deprivation/reoxygenation (OGD/R) cell model were constructed. THRIL was knocked down using siTHRIL. Neurological deficit score was detected based on the criteria of Zea-Longa. Brain region 2,3,5-Triphenyltetrazolium (TTC) staining and quantitative analysis of cerebral infarction volume, RT-qPCR, and fluorescence immunostaining were performed for assessing THRIL expression. MTT assay was used to detect the cell proliferation ability after transfection, TUNEL assay was applied to detect apoptosis, and western blot and ELISA detected related protein expression. A dual luciferase reporter system and RIP assay were used to confirm the target relationship.

Results: THRIL was upregulated in both *in vitro* and *in vivo* models of brain ischemia-reperfusion injury. Knockdown of THRIL attenuated OGD/R neuronal apoptosis and OGD/R-induced inflammation. THRIL targeted and regulated the expression of miR-24-3p/neuropilin-1 (NRP1) axis. THRIL silencing significantly improved the neurological functioning of rats in the MCAO/R model by miR-24-3p/NRP1/NF-κB p65 signaling pathway.

Conclusion: THRIL could aggravate cerebral ischemia-reperfusion injury by competitively binding to miR-24-3p to promote the upregulation of NRP1 and further promoted the activation of the NF-κB p65 signaling pathway.

## INTRODUCTION

The brain is the most sensitive organ for ischemia and hypoxia [1], and cerebral ischemia can lead to necrosis or apoptosis of the brain cells [2]. Timely thrombolysis in the treatment time window and rapid and effective reconstruction of the collateral circulation of the microvessels to restore blood reperfusion in the ischemic region the and penumbra is the best treatment for cerebral ischemia; however, blood flow after ischemia recanalization may lead to ischemia-reperfusion injury [3]. The principle of treatment for ischemic cerebrovascular disease is to restore blood perfusion in the ischemic area in time [4]. However, after reperfusion, the dysfunction and structural damage caused by ischemia are further aggravated, so inhibition of reperfusion injury has become an important part of treatment [5]. Recently, the treatment of cerebral ischemia-reperfusion injury has achieved a degree of progress, and many therapeutic targets have been identified.

Tumor necrosis factor (TNF) and HNRNPL related immunoregulatory lncRNA (THRIL) is a novel long non-coding (lnc) RNA that plays a key role in the regulation of TNF-α expression [6]. Overexpression of THRIL may aggravate HKS cell injury induced by lipopolysaccharide (LPS) through regulating the microRNA miR-34a expression, thereby preventing the degradation of MCP-1 [7]. In addition, knockdown of THRIL participates in the progression of myocardial infarction by regulating miR-99a to protect against hypoxia-induced H9C2 cell injury [8]. In osteoarthritis, THRIL promotes LPS-induced inflammatory damage by downregulating miRNA-125b in ATDC5 cells [9]. The expression of THRIL is increased in T cells of patients with rheumatoid arthritis, and THRIL plays a key role in adaptive immune cell differentiation and functions [10]. Liu et al. reported that LncRNA THRIL was upregulated in sepsis and sponged miR-19a to upregulate TNF-alpha expression, and promoted lung cell apoptosis [11]. These studies fully explain that THRIL plays a crucial role in cell apoptosis and inflammation. Through our preliminary experiments, we found that THRIL was up-regulated *in vitro* and *in vivo* models of brain ischemia-reperfusion injury. Thus, we focused on the role of THRIL in the apoptosis and inflammation during the the cerebral ischemia-reperfusion injury. Our aim of this study was to investigate the role and the molecular mechanism of THRIL in cerebral ischemia-reperfusion injury.

Based on the role of THRIL in inflammation-related diseases, we first studied the role of THRIL in cerebral ischemia-reperfusion injury. The RNA22 prediction site (https://cm.jefferson.edu/rna22/Interactive/) predicted that THRIL could have a potential binding site with miR-24-3p, and further experimental validation confirmed this targeting. As shown in previous studies, miR-24-3p inhibits the development of aortic vascular inflammation and mouse celiac aneurysm, and also reduces myocardial apoptosis and modulates the KEAP1-NRF2 pathway in ischemia-reperfusion injured rats [12]. Myocardial ischemia-reperfusion injury was reduced by inhibiting the expression of RIPK1 in rats [13, 14]. The above results showed that this could inhibit ischemia-reperfusion injury and inflammation. A study has also indicated that rosuvastatin reduces myocardial ischemia-reperfusion injury by downregulating hsa-miR-24-3p and targeting up-regulated decoupling protein 2 [15]. However, the role of miR-24-3p in cerebral ischemia-reperfusion injury is unclear. In this study, using online prediction tools, a total of 85 target genes that can combine with miR-24-3p were obtained. Among them, the target gene neuropilin-1 (*NRP1*) is a direct target of the transcription factor E2F1 in cerebral ischemia-induced neuronal death [16]. This target gene directly interacts with Fer kinase, mediates semaphorin 3a-induced cortical neuron death, and promotes cerebral ischemia-reperfusion injury [17]. NRP1 has been confirmed to promote p65 signaling pathway activation [18]. Our study demonstrates that THRIL can aggravate cerebral ischemia-reperfusion injury by competitively binding to miR-24-3p to promote the upregulation of NRP1 expression and further promote the activation of the NF-κB p65 signaling pathway.

## RESULTS

### THRIL is upregulated in the *in vitro* and *in vivo* models of brain ischemia-reperfusion injury

**Figure 1:**
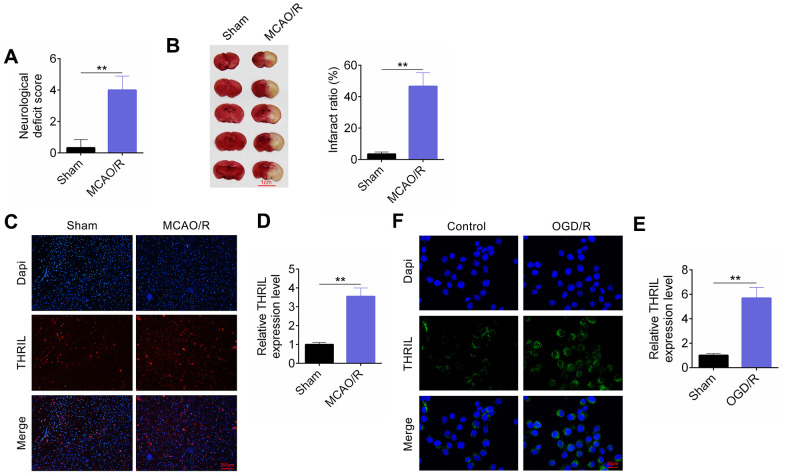
**LncRNA THRIL is up-regulated *in vitro* and *in vivo* models of brain I/R injury.** (**A**) The neurological deficit score. (**B**) The infract ratio. (**C**) Fluorescent immunostaining assay was performed *in vivo* models. (**D**) RT-qPCR assay detected lncRNA THRIL expression *in vivo* models. (**E**) Fluorescent immunostaining assay *in vitro* model. (**F**) RT-qPCR assay detected lncRNA THRIL *in vitro* model. Data are shown as mean ± SD for three-independent experiments. **P < 0.01.

The neurological deficit score in the MCAO/R group was significantly higher than that in the sham group (Figure 1A). Moreover, the infarct ratio in the MCAO/R group was also higher than that in the control group (Figure 1B), which confirmed that the MCAO/IR rat model had been successfully constructed. Based on the results of the fluorescent immunostaining and RT-qPCR assays, there was greater expression of THRIL in the MCAO/R and OGD/R models (Figure 1C–1F, P < 0.01). In addition, THRIL was mainly localized in the cytoplasm (Figure 1C, 1E).

### Knockdown of THRIL reduces apoptosis of OGD/R neurons

**Figure 2:**
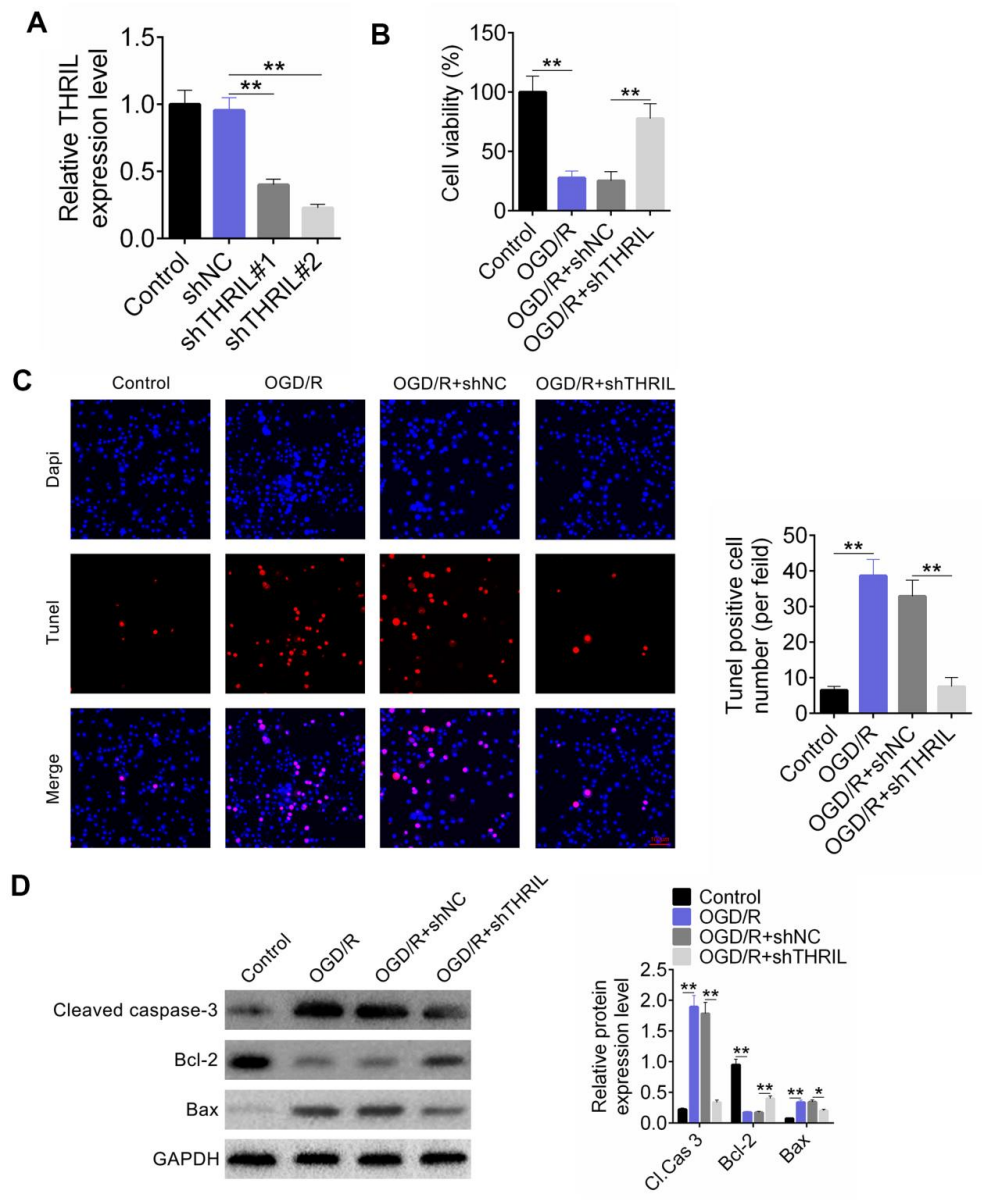
**Knockdown of lncRNA THRIL reduces apoptosis of OGD/R neurons.** (**A**) Transfection efficiency of shTHRIL. (**B**) Cell viability was examined by CCK-8. (**C**) The apoptosis of OGD/R was exaimed by TUNEL assay. (**D**) Western blot assay detected the expressions of cleaved caspase-3, Bcl-2, and Bax. Data are shown as mean ± SD for three-independent experiments. **P < 0.01.

Based on the transfection, the OGD/R neurons were divided into four groups: control, sh-NC, shTHRIL#1, and shTHRIL#2 groups and the transfection efficiency of shTHRIL#1 and shTHRIL#2 was quantified by RT-qPCR. Both shTHRIL shRNAs significantly decreased the expression of THRIL compared to sh-NC group, which confirmed that the transfection was successful. In addition, shTHRIL#2 exhibited higher transfection efficiency than shTHRIL#1 (Figure 2A). Therefore, shTHRIL#2 was used for the subsequent experiments and named shTHRIL. The cell viability of OGD/R cells was significantly lower than that of the control (P < 0.01). After transfection with shTHRIL, the viability of OGD/R cells increased (Figure 2B). Interestingly, TUNEL-positive cell numbers in the OGD/R and OGD/R+shNC groups were significantly greater than those in the control and OGD/R+shTHRIL groups (Figure 2C). Western blot analysis detected the expression of apoptosis-related proteins. Similarly, the expression of cleaved-caspase3 (CASP-3) and BAX was upregulated in the OGD/R and OGD/R+shNC groups, whereas shTHRIL caused a clear decrease in expression. The expression of Bcl-2 was negatively correlated with cleaved-CASP-3 and BAX (Figure 2D).

### Knockdown of THRIL reduces OGD/R-induced inflammation

**Figure 3:**
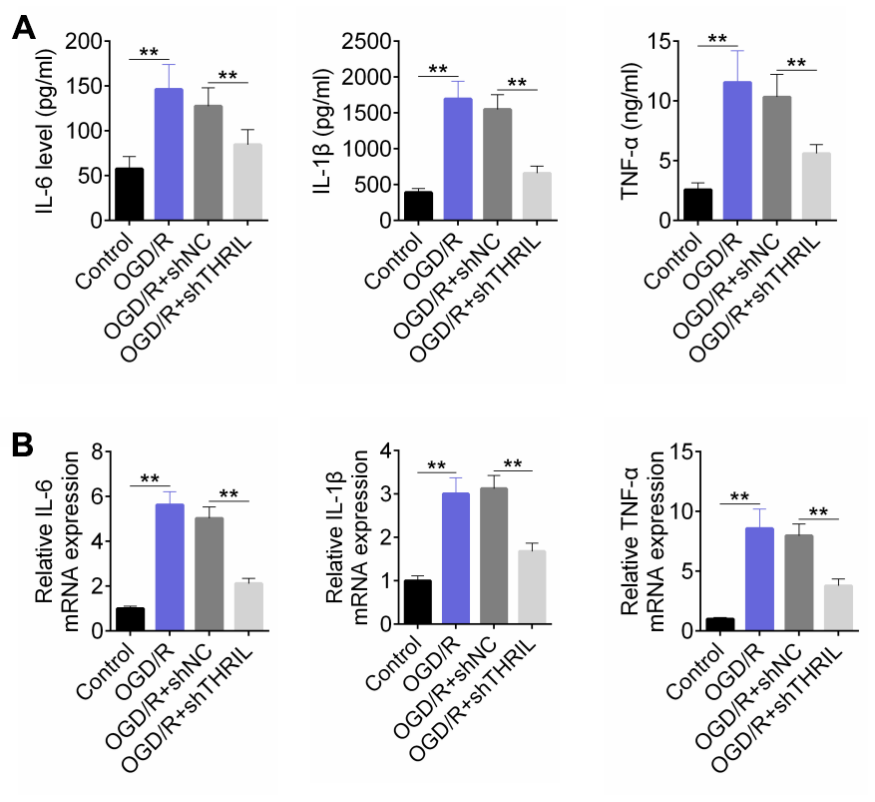
**Knockdown of lncRNA THRIL reduces OGD/R-induced inflammation.** (**A**) ELISA assay for the concentration of inflammation factors, including IL-6, IL-1β and TNFα. (**B**) RT-qPCR detected the expressions of IL-6, IL-1β and TNFα. Data are shown as mean ± SD for three-independent experiments. **P < 0.01**.**

Transfection of shTHRIL was performed in human neuroblastoma SH-SY5Y cells, followed by the detection of the levels of IL-6, IL-1β, and TNFα. Based on the treatment, the cells were divided into the control, OGD/R, OGD/R+shNC, and OGD/R+shTHRIL groups. The concentrations of inflammatory factors, including IL-6, IL-1β, and TNFα, were significantly higher in the OGD/R and OGD/R+shNC groups (Figure 3A), whereas shTHRIL clearly decreased the levels of these factors. RT-qPCR results reflected this tendency (Figure 3B).

### THRIL targets and regulates the expression of miR-24-3p

**Figure 4:**
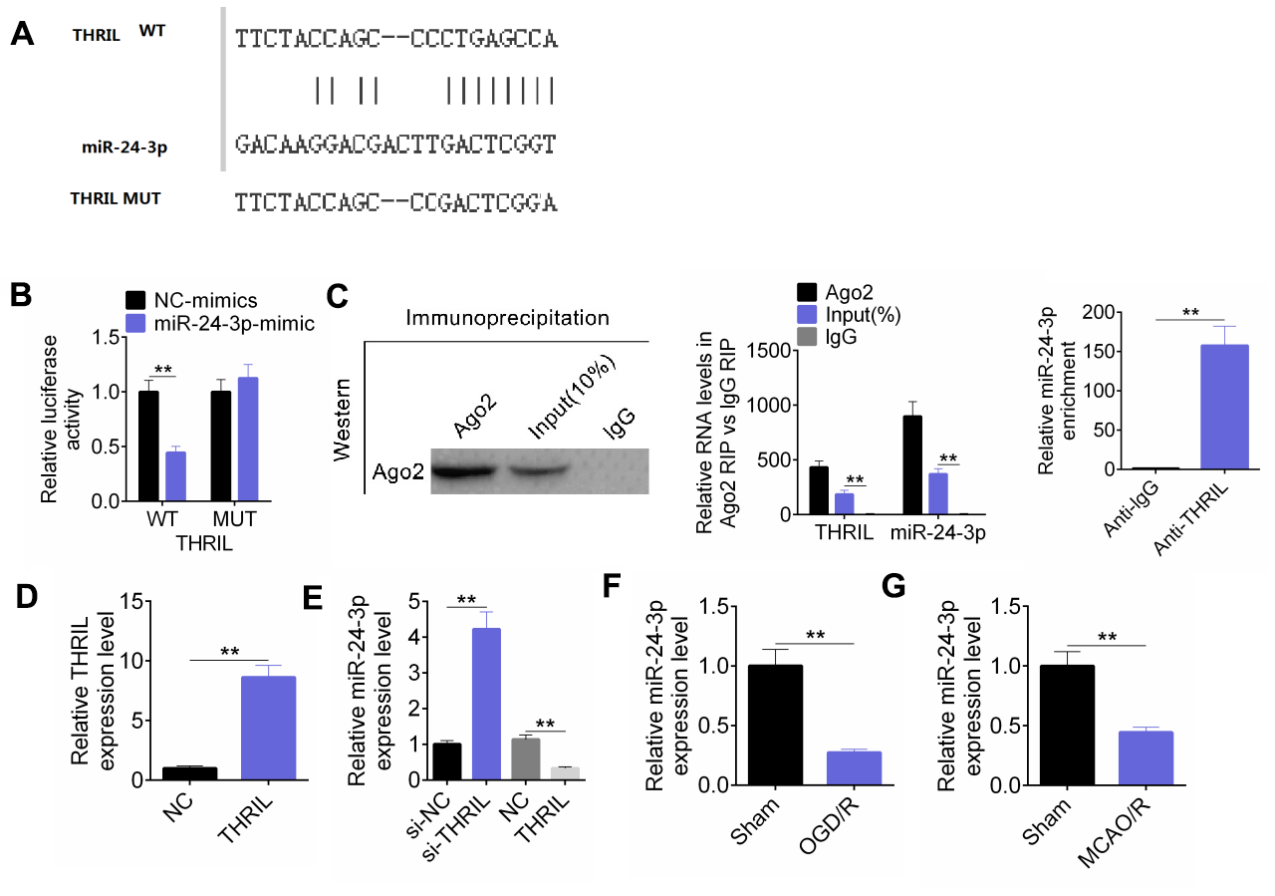
**LncRNA THRIL targets and regulates the expression of miR-24-3p.** (**A**) The binding site was predicted. (**B**) The dual luciferase reporter assay was performed to verify the combination of THRIL and miR-24-3p. (**C**) RIP experiment was performed to prove the combination of THRIL and miR-24-3p. (**D**) RT-qPCR detected THRIL overexpression efficiency. (**E**) RT-qPCR detected the expression of miR-24-3p in SH-SY5Y cells with THRIL knockdown or overexpression. (**F**) RT-qPCR detected the expression of miR-24-3p in OGD/R models. (**G**) RT-qPCR detected mRNA levels of miR-24-3p in MCAO. Data are shown as mean ± SD for three-independent experiments. **P < 0.01.

To explore the regulatory mechanism involved, the binding site was predicted, as shown in Figure 4A. The fluorescence intensity of THRIL was measured using a dual luciferase reporter system. The fluorescence intensity of the pmirGLO-THRIL-wt+ miR-24-3p- mimic group decreased, and the luciferase intensity of the pmirGLO-THRIL-mut group increased (Figure 4B). The amount of THRIL bound to Ago2 or IgG after the RIP experiment was examined. miR-24-3p was enriched by THRIL, indicating that THRIL binds to miR-24-3p (Figure 4C). RT-qPCR detected the expression levels of THRIL and miR-24-3p. As shown in Figure 4D, THRIL was overexpressed in SH-SY5Y cells. Overexpression of THRIL resulted in the downregulation of miR-24-3p expression, whereas knockdown of THRIL induced the upregulation of miR-24-3p expression (Figure 4E). RT-qPCR analysis of the mRNA levels of miR-24-3p in MCAO and OGD/R models demonstrated that the relative miR-24-3p expression level was significantly lower in the MCAO and OGD/R models than that in the sham group (Figure 4F, 4G).

### NRP1 was the target of miR-24-3p

**Figure 5:**
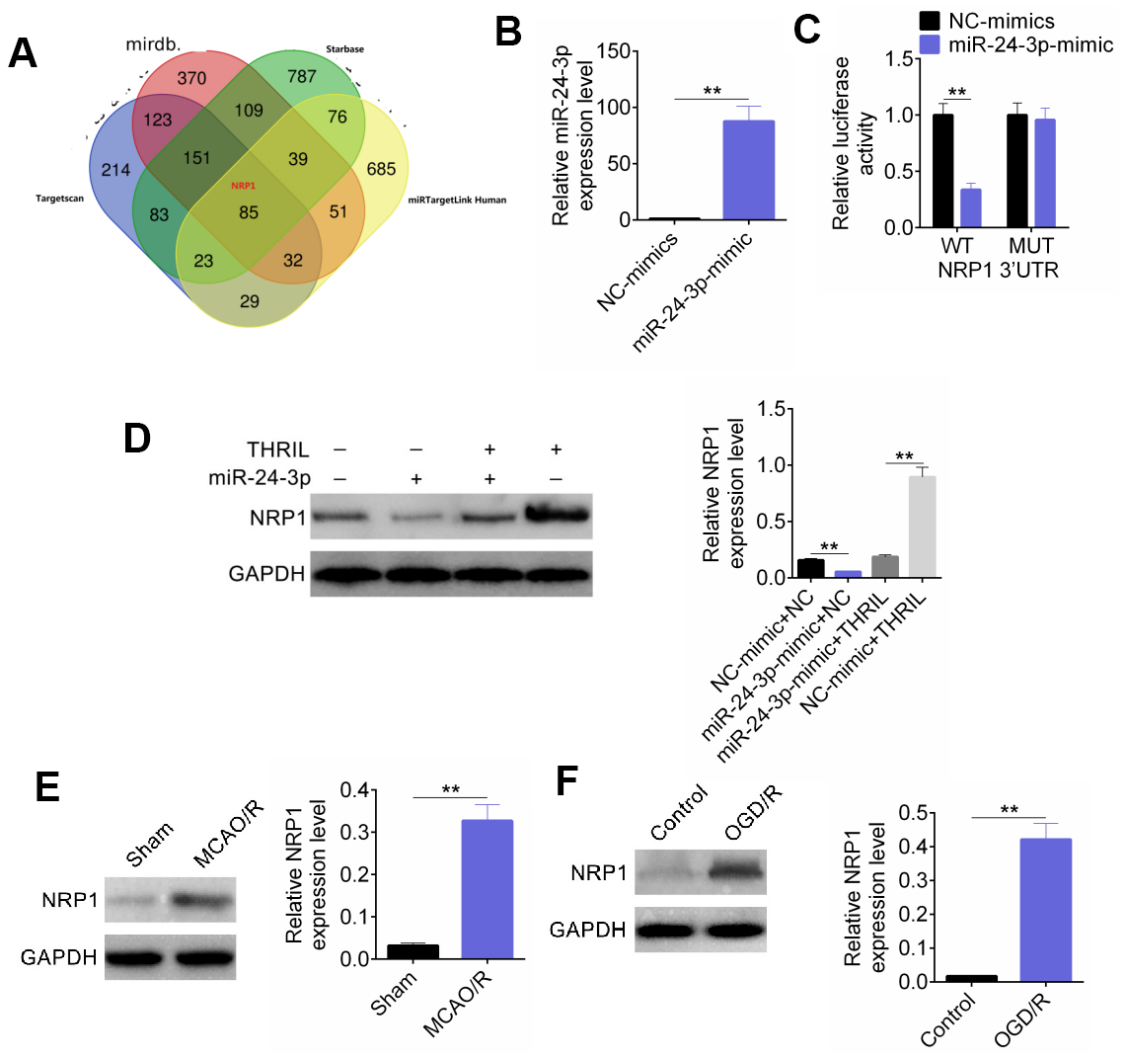
**NRP1 was the target of miR-24-3p.** (**A**) The binding site of NRP1 and miR-24-3p. (**B**) RT-qPCR was used for detection of miR-24-3p transfection efficiency. (**C**) The fluorescence intensity of NRP1 3'-UTR was detected by dual luciferase reporter assay. (**D**) Western blot detected the expression of NRP1. (**E**) The expression of NRP1 was significantly higher in MCAO model. (**F**) The expression of NRP1 was significantly higher in OGD/R model. Data are shown as mean ± SD for three-independent experiments. **P < 0.01.

Based on the forecasting website (miRDB, http://www.mirdb.org/; miRTargetLink Human, https://ccb-web.cs.uni-saarland.de/mirtargetlink/network.Php?type=Target_Gene&qval=ITGA8; Targetscan, http://www.targetscan.org/vert_71/; Starbase, http://starbase.sysu.edu.cn/starbase2/index.php), we predicted target genes that bind to miR-24-3p and obtained 85 target genes that can be bound. Among them, the target gene neuropilin-1 (NRP1) was a direct target of the transcription factor E2F1 in cerebral ischemia-induced neuronal death (Figure 5A). RT-qPCR showed that miR-24-3p transfection was successful (Figure 5B). A dual luciferase reporter system was used to detect the fluorescence intensity of the NRP1 3′-UTR. The relative luciferase activity of NRP1 in NC-mimic-WT was significantly lower than that in the four groups (Figure 5C). Based on the transfection, the cells were divided into NC-mimic + pcDNA3.1-NC, miR-24-3p-mimic + pcDNA3.1-NC, miR-24-3p-mimic+ pcDNA3.1-THRIL, and NC-mimic + pcDNA3.1-NC-THRIL groups. The expression of NRP1 protein was detected by western blotting, which showed that miR-24-3p significantly decreased the expression of NRP1, whereas THRIL could reverse this effect. In addition, western blot analysis showed that the expression of NRP1 was significantly higher in the MCAO and OGD/R models than that in the control (Figure 5E, 5F).

### THRIL regulates NF-κB p65 signaling pathway through miR-24-3p and further regulates OGD/R-induced neuronal apoptosis and inflammatory response

**Figure 6:**
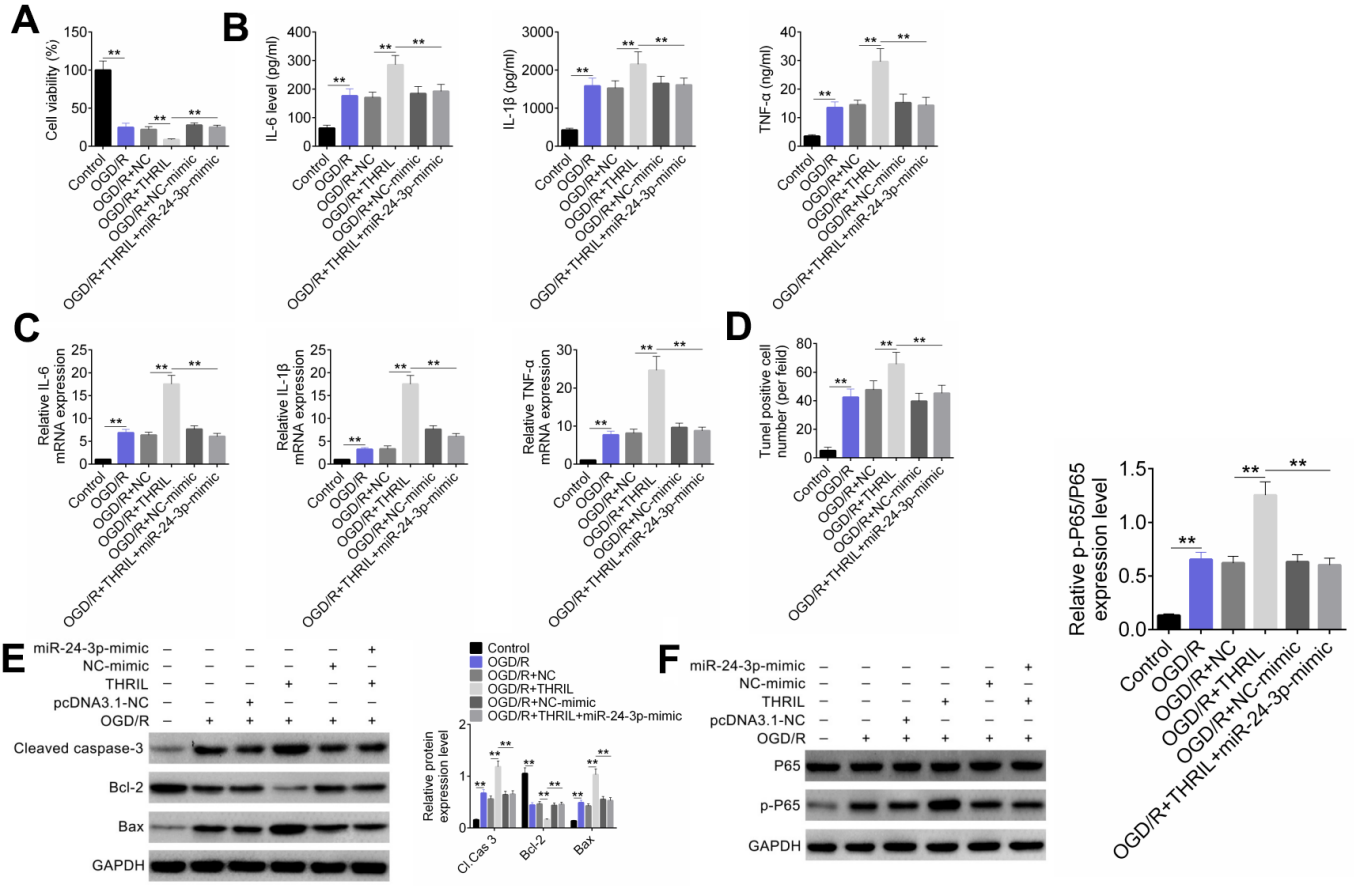
**LncRNA THRIL regulates NF-κB p65 signaling pathway through miR-24-3p, and further regulates OGD/R-induced neuronal apoptosis and inflammatory response.** (**A**) SH-SY5Y cells were transfected and divided into six groups: control, OGD/R, OGD/R+pcDNA3.1-NC, OGD/R+pcDNA3.1-THRIL, OGD/R+NC-mimic, and OGD/R+pcDNA3.1-THRIL+miR-24-3p-mimic groups. (**A**) Cellular activity was identified by CCK-8. (**B**) The concentrations of inflammatory factors (IL-6, IL-1β, TNFα) were examined by ELISA. (**C**) The expression levels of inflammatory factors (IL-6, IL-1β, TNFα) were detected by RT-qPCR. (**D**) TUNEL was performed to evaluate cell apoptosis. (**E**) The expression levels of apoptotic proteins were detected by western blot. (**F**) The p65 protein expression was detected by western blot. Data are shown as mean ± SD for three-independent experiments. **P < 0.01.

To investigate the role of THRIL and miR-24-3p in neuronal apoptosis and inflammatory response, cells were transfected and divided into six groups: control, OGD/R, OGD/R+pcDNA3.1-NC, OGD/R+ pcDNA3.1-THRIL, OGD/R+NC-mimic, and OGD/R+pcDNA3.1-THRIL+miR-24-3p-mimic groups. Cellular activity, apoptosis, apoptotic protein, inflammatory factors (IL-6, IL-1β, and TNFα), and p65 protein expression were detected. The results showed that in the OGR/D model, the cell viability was significantly decreased in comparison to the control group, and THRIL further decreased the effect, whereas the miR-24-3p-mimic rescued this effect and increased the viability (Figure 6A). The levels of inflammatory factors (IL-6, IL-1β, TNFα) were highest in the OGD/R+ pcDNA3.1-THRIL group, and miR-24-3p-mimic could also rescue the inflammatory effect of THRIL (Figure 6B); the RT-qPCR assay showed the same tendency as the ELISA assay (Figure 6C). The TUNEL assay was used to detect the apoptotic cell ratio. Similarly, the TUNEL-positive cell number was the largest in the OGD/R+ pcDNA3.1-THRIL group. miR-24-3p-mimic rescued this effect and decreased the number of apoptotic cells (Figure 6D). Western blot analysis was used to detect the expression of inflammatory factors (IL-6, IL-1β, and TNFα) and p65 protein. The expression of CASP-3, the ratio of BAX/Bcl-2, and the relative p-p65/p65 expression were significantly higher in the OGD/R+ pcDNA3.1-THRIL group, whereas miR-24-3p-mimic rescued this effect (Figure 6E, 6F). Therefore, THRIL regulates the NF-κB p65 signaling pathway through miR-24-3p, and further regulates OGD/R-induced neuronal apoptosis and inflammatory response.

### Silencing of THRIL significantly improved the neurological function of the rat MCAO/R model

**Figure 7:**
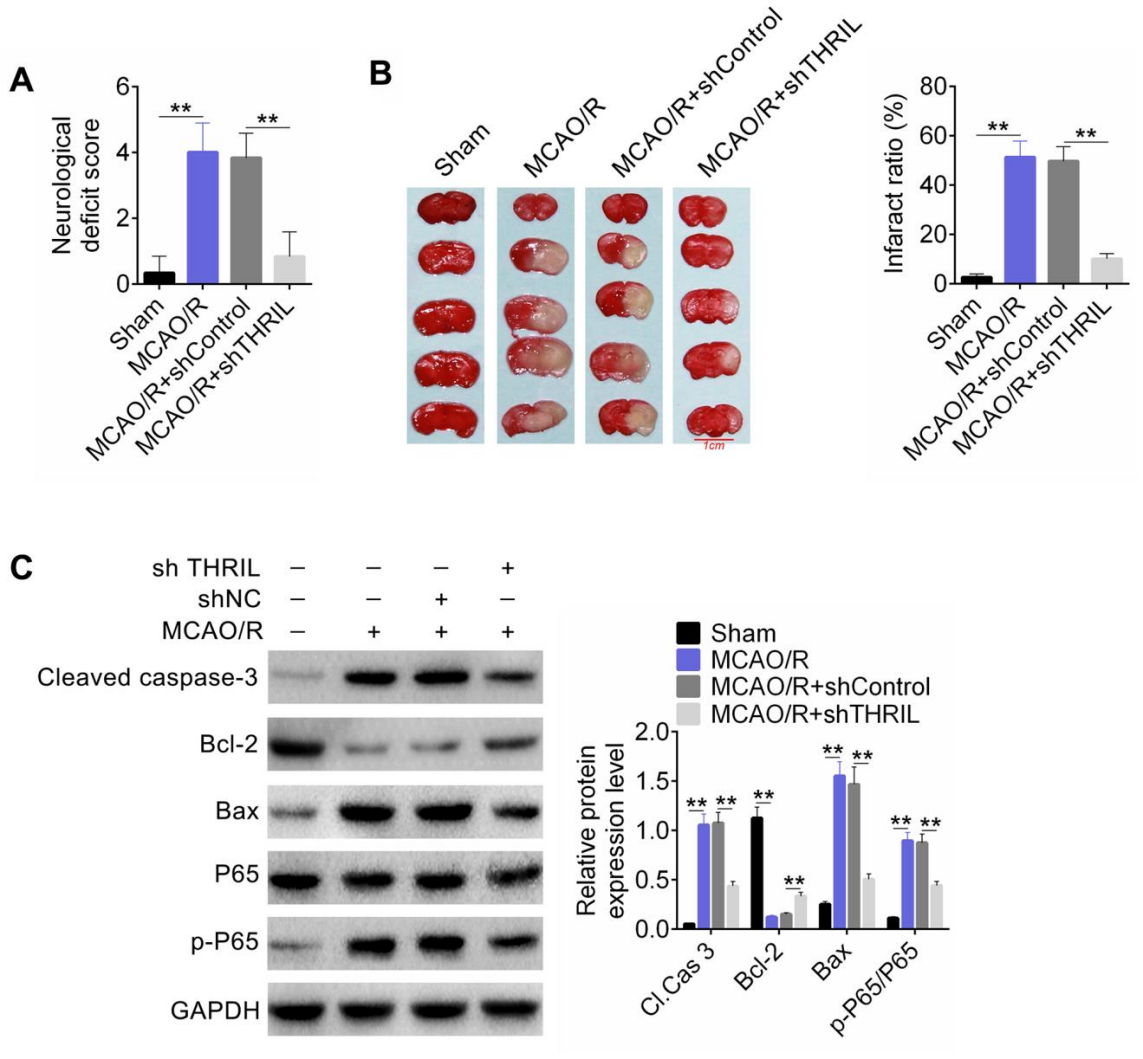
**LncRNA THRIL silencing significantly improved the neurological function of the rat MCAO/R model.** (**A**) Nerve injury score of the rat MCAO/R model. (**B**) Cerebral infarct size was assessed by TTC staining. (**C**) Western blot detected the apoptosis and p65-associated proteins. Data are shown as mean ± SD for three-independent experiments. **P < 0.01.

The MCAO/R rat model was divided into four groups: sham, MCAO/R, MCAO/R+sh control, and MCAO/R+sh THRIL groups. Nerve injury scoring, cerebral infarct size, western blot detection of apoptosis, and p65-associated protein were assessed for the above groups. As shown in Figure 7A, the neurological deficit score was significantly higher in the MCAO/R+sh control group, whereas silencing of THRIL significantly decreased this score. In addition, THRIL silencing significantly reduced the infract ratio in the MCAO/R model (Figure 7B). In the MCAO/R model, the relative protein expression levels of CASP-3, the ratio of BAX/Bcl-2, and p-p65/p65 were significantly increased, whereas silencing of THRIL decreased their expression (Figure 7C). Therefore, THRIL silencing significantly improved the neurological function of the rat MCAO/R model.

## DISCUSSION

The incidence of ischemic cerebral vascular disease is increasing annually, and the mortality rate is as high as 60% to 80% [19]; survivors may also experience serious impacts on their quality of life [20]. In this study, we first investigated the role of THRIL in cerebral ischemia-reperfusion injury. We demonstrated that THRIL was upregulated in both *in vitro* and *in vivo* models of brain ischemia-reperfusion injury. Knockdown of THRIL attenuated OGD/R neuronal apoptosis and OGD/R-induced inflammation. We also showed that THRIL targeted and regulated the expression of miR-24-3p, and NRP1 was confirmed to be a target gene of miR-24-3p using online prediction tools and inflammation responses. Furthermore, THRIL regulated the NF-κB p65 signaling pathway through miR-24-3p to affect the OGD/R-induced neuronal apoptosis and inflammatory response. Silencing of THRIL significantly improved the neurological functioning of rats in the MCAO/R model.

*THRIL* is an RNA gene, and is affiliated with the non-coding RNA class [21]. In multiple sclerosis patients, THRIL has been closely related with Fas cell surface death receptor-antisense 1 (FAS-AS1), playing a key role in the regulation of immune responses [22]. In addition, THRIL has been confirmed to interact with hnRNPL, and then regulate the expression of TNF-α, a finding that may reflect a critical progress in our knowledge about the inflammatory and immune responses in humans [23]. Neutrophils have been shown to adhere to ischemic vascular endothelial cells when blood flow velocity is reduced during cerebral ischemia, initiating an acute inflammatory reaction [24]. After reperfusion, the blood carries neutrophils from other sites to the cerebral ischemic site where they accumulate [25]. This can impede brain microcirculation, causing effects such as brain edema and cell necrosis, which in turn could lead to cerebral infarction [26]. Microglia are considered to be potential macrophages in the brain and closely related to the inflammatory response of CIRI [27]. It can be activated a few minutes after cerebral ischemia and is converted into brain macrophages when brain cells appear to be apoptotic. Interestingly, downregulation of THRIL could decrease hypoxia-induced injury in rat cardiomyocytes [28]. Similar results were obtained in this study, where THRIL was upregulated in models of brain ischemia-reperfusion injury, which was closely related to OGD/R-induced inflammation. Therefore, THRIL might improve brain ischemia-reperfusion injury by participating in inflammation.

Notably, THRIL could inhibit the expression of miR-24-3p in this study. As reported in previous studies, miR-24-3p could regulate the expression of MXI1 and improve the proliferation of glioma cells [29, 30]. More importantly, in this study, THRIL promoted the upregulation of NRP1 by competitively binding miR-24-3p. NRP1 has been shown to play an important role in axonal growth and neuronal death induced by cerebral ischemia [31]. Interestingly, semaphorin-3a binds to its receptor, NRP-1, to form a receptor complex and is involved in the regulation of axon guidance, branching, bunching, and synapse formation [32]. Fujita et al. [33] found that the expression of Sema 3a and NRP-1 was upregulated in the ischemic cerebral cortex after ischemic brain injury. Shirvan et al. [34] confirmed that after ischemic cerebral infarction, apoptotic neurons produced and secreted Sema 3a, which induced the apoptosis and necrosis of adjacent neuronal cell populations. This induction could be blocked by antibodies to NRP-1. Therefore, NRP-1 is involved in the regulation of neuronal survival and growth, axonal repair, and regeneration after ischemic brain injury.

In this study, the upregulation of NRP1 expression in cerebral ischemia-reperfusion rats further promoted the activation of the NF-κB p65 signaling pathway. Exaggerated activation of NF-κB p65 has been confirmed to improve the immune mechanism of necrosis and reperfusion injury [35]. Similarly, NF-κB expression is related to rat brain damage, and inhibition of the NF-κB signaling pathway is an important means to reduce cerebral ischemia-reperfusion injury [36]. Xue et al. [37] showed that baicalin could inhibit NF-κB activation and then extenuate focal cerebral ischemic reperfusion injury. In this study, THRIL silencing significantly improved the neurological functioning of the rats in the MCAO/R model. Thus, THRIL aggravates cerebral ischemia-reperfusion injury.

In conclusion, THRIL could aggravate cerebral ischemia-reperfusion injury by competitively binding to miR-24-3p to promote the upregulation of NRP1 and further promote the activation of the NF-κB p65 signaling pathway. Therefore, THRIL may be a novel target for the prediction and treatment of cerebral ischemia-reperfusion injury.

## MATERIALS AND METHODS

### Construction of middle cerebral artery occlusion/ischemia-reperfusion (MCAO/IR) rat model and tissue collection

Twenty-five male Sprague-Dawley rats weighing 223 ± 18 g were provided by the Animal Experimental Center of Southern Medical University. In addition, five THRIL-knockdown rats were purchased from Beijing Vitalstar Biotechnology Co., Ltd. This study was approved by the Animal Ethics Committee of the First Affiliated Hospital of Soochow University. All SD rats were housed in a laboratory barrier environment at room temperature (23 ± 3° C) with free access to water; relative humidity was maintained at 57 ± 11%. Animals were fasted for 12 h before surgery but were free to drink water. They were randomized into sham and MCAO/R groups. Rats in the MCAO/R group were anesthetized with 10% chloralhydrate at a dose of 3 mL/kg. A midline incision in the neck was made to separate the skin and muscles in sequence, exposing the right common carotid artery and its branching internal and external carotid arteries. The valgus nerve associated with the common carotid artery was carefully separated, and the common carotid artery and the proximal end of the external carotid artery were ligated, and the internal carotid artery was alive. A small opening was made at the distal end of the right common carotid artery. The line was inserted from the right common carotid artery into the middle cerebral artery through the internal carotid artery. After 2 h, the line plug was removed. For the sham group, rats were fully anesthetized and a median neck incision was made. The external carotid artery, internal carotid artery, and common carotid artery were isolated but not ligated, and the incision was closed 30 min later. After reperfusion for 24 h, approximately 5 mL of blood was taken from the abdominal aorta after deep anesthesia. Subsequently, the rats were euthanized by spine dislocation, and the ischemic penumbra of the rat cerebral cortex was placed in liquid nitrogen for storage.

### Construction of OGD/R cell model

SH-SY5Y cells were cultured in DMEM medium containing 10% fetal bovine serum, 100 kU/L penicillin and 100 mg/L streptomycin in 5% CO_2_, 37° C incubator, and changed every other day. SH-SY5Y cells in the logarithmic growth phase were collected and divided into sham and OGD/R groups. For glucose-oxygen deprivation, the media for the OGD/R group was replaced following cell attachment with a preheated glucose-free balanced salt solution (pH 7.4), and the cells were incubated in a mixture containing 1% O_2_, 5% CO_2_, and 94% N_2_, at 37° C saturated temperature and humidity. The initial O_2_ concentration was 5 L/min. After 30 min, the O_2_ concentration in the incubator was reduced to 1%, and the oxygen-deficient gas mixture was continuously filled. The O_2_ concentration in the incubator was maintained below 1%, and the sugar and oxygen deprivation was continued for 6 h. The media was then replaced with DMEM and reperfused for 12 h in a 37° C saturated humidity incubator with 5% CO2.

### Transfection

Two shRNA plasmids (pcDNA3.1 vector) were designed and purchased from Vigene Biosciences, based on the different regions of the THRIL sequence. The SH-SY5Y cells and OGD/R cells in the logarithmic growth phase were added to a 6-well plate at 3×10^6^ cells/mL, 100 μL per well, and cultured overnight. The transfection reagent Lipofectamine 2000 was used to transfect shRNA plasmid 1 and THRIL shRNA plasmid 2. Then, the cells were cultured at 37° C with saturated humidity for 48 h for subsequent experiments. Untransfected cells were used as a control group. Similarly, miR-24-3p-mimic, NC mimic, si-THRIL, and the overexpression vector pcDNA3.1-THRIL were also transfected as described above.

### Neurological deficit score

Neurological deficit score was detected based on the criteria of Zea-Longa as follows: 0 points, no symptoms of nerve defects; 1 point, rats could not fully extend the contralateral forelimb; 2 points, the body turned to the hemiplegia side while walking; 3 points, the body leaned to the hemiplegia side when walking; 4 points, the rat could not be self-issued with loss of consciousness. Neurological deficit scores were scored 24 h after reperfusion.

### Determination of cerebral infarction volume

Six hours after ischemia-reperfusion, anesthetized animals were intraperitoneally injected with 10% chloral hydrate (0.3 mL/100 g body weight), and the brain was removed and immediately stored at -20° C for 20 min. The sample was continuously coronally sliced into a brain slice mold with a sheet thickness of 2 mm. The brain slices were transferred to a 2% TTC (triphenyltetrazolium chloride) solution (0.1 mol/L PBS, pH 7.4) and incubated at 37° C for 30 min at a constant temperature. Brain tissue sections were photographed and analyzed using the Luzex-F image analysis system, and the integral method was used to calculate the infarct ratio.

### Immunofluorescence assay

After the slide was washed three times with 0.01 mol/L PBS, Triton X-100 solution was added for 30 min (37° C). After blocking endogenous peroxidase with 0.3% H_2_O_2_, 1:100 rabbit anti-rat THRIL antibody (Santa Cruz Inc.) was added dropwise, and the slide was incubated at room temperature for 1 h and then placed in a refrigerator at 4° C overnight. After rewarming for 1 h on the second day, fluorescein-labeled TdT reaction solution and rhodamine-labeled goat anti-rabbit IgG were added to the sample for 60 min at 37° C, which was then washed with 0.01 mol/L PBS three times. A fluorescence microscope (Olympus BX51-DP70) was used to observe and photograph images. For OGD/R cells and sham cells, the cells were seeded in a 6-well plate containing a coverslip at 3 to 5 × 10^5^ per well. After the cells had attached for 48 h, the coverslips were collected, washed three times with PBS, and fixed in 40 g/L paraformaldehyde for 30 min at room temperature. The coverslips were removed, fixed on glass slides, and washed three times with PBS. The other steps were the same as above.

### Reverse transcriptase-quantitative (RT-q) PCR assay

Total RNA from tissue samples and cells was extracted by rapid extraction using TRIzol reagent. After DNase treatment, cDNA was reverse transcribed into cDNA, and the synthesized cDNA was stored in a refrigerator at -20° C until use. The RT-qPCR reaction conditions were as follows: pre-denaturation at 95° C for 30 s, 95° C for 5 s, 63° C for 30 s, and 95° C for 10 s for 40 cycles. The primer sequences were as follows: THRIL, (upstream) 5’-CTCCAGGAGAGTTTGGGTCC-3’, (downstream) 5’-AGATAACCCTGCCAGACCT-3’; IL-6, (upstream) 5’-CCACTGCCTTCCCTACTTCA-3’, (downstream) 5’-AACGGAACTCCAGAAGACCA-3’; IL-1β. (upstream) 5’-TGAAGCAGCTATGGCAACTG-3’, (downstream) 5’-AAAGAAGGTGCTTGGGTCCT-3’; TNFα, (upstream) 5’-CAGCAGATGGGCTGTATCTT-3’, (downstream) 5’-AAGTAGACCTGCCCGGACTC-3’.

### CCK-8 assay

The transfected cells were inoculated into 96-well microplates at 2×10^3^ cells/mL, 100 μL per well, and five replicate wells were set in each group. The cells were cultured overnight to adhere and then cultured for 72 h for use in the CCK-8 assay. CCK-8 reagent (10 μL) was added to each well and the incubation was continued for 2 h. Finally, the optical density (OD) value at 450 nm was measured using a microplate reader.

### TUNEL assay for apoptosis detection

Paraffin sections were prepared using conventional methods. According to the TUNEL Apoptosis Detection Kit (Sigma-Aldrich), the samples were washed three times with phosphate buffer and soaked in methanol at -20° C for 15 min. Samples were then washed three times with phosphate buffer and covered with a balanced salt buffer for 30 min. The tissue was covered with the reaction complex, incubated at room temperature for 60 min, washed with phosphate buffer three times, and then sealed. Finally, a fluorescence analyzer was used for analysis.

### Western blot assay

The total cell protein solution was extracted. After measuring the protein concentration using the BCA protein quantification kit, 60 μg of protein was taken from each group, and an appropriate amount of 5× protein loading buffer was added and the solution was placed in a boiling water bath for 5 min. A 10% separation gel and a 5% stacking gel were used to separate the proteins, and then the separated protein was transferred to a PVDF membrane. The membranes were blocked with 5% skim milk powder (containing 0.5% TBST) for 1 h. Primary antibodies (TBST dissolved 5% skim milk, phosphorylated protein, 5% BSA dissolved in TBST) (rabbit anti-BAX monoclonal primary antibody 1:2000, rabbit anti-Bcl-2 monoclonal primary antibody 1:1000, rabbit anti-cleaved-caspase3 monoclonal primary antibody 1:250, NRP1 antibody 1:1000) were added and with overnight incubation at 4° C. After washing with TBST, a diluted secondary antibody (TBST-dissolved 5% skim milk) (HRP goat anti-rabbit IgG secondary antibody 1:10000) was added, and the membrane was incubated for 30 min at room temperature, and then washed three times with TBST at room temperature. The exposure was developed using the ECL method. X-ray film was used for exposure and development. The Image J software processing system was used to analyze the optical density values of the target bands. GAPDH was used as an internal reference.

### Enzyme-linked immunosorbent assay (ELISA)

ELISA was used to detect the concentrations of IL-1β, TNF-α, and IL-6 proteins. The procedure was in accordance with the kit instructions. After the reaction was terminated, the enzyme labeling plate was placed in the microplate reader, and the absorbance at 450 nm was measured. The standard product and the blank control area were determined, and the standard curve was automatically detected and calculated by the enzyme labeling detector.

### Dual luciferase reporter assay

The THRIL gene wild-type and mutant dual luciferase reporter vectors were constructed. 293T cells were cultured in DMEM high sugar medium containing 10% fetal bovine serum. 293T cells were trypsinized, washed with PBS, and then adjusted with 1.4 mL PBS to adjust the cell density to 2 × 10^7^ mL. Based on the transfection, the cells were divided into four groups: pmirGLO-THRIL-wt+NC mimic, pmirGLO-THRIL-wt+miR-24-3p-mimic, pmirGLO-THRIL-mut+NC mimic, and pmirGLO-THRIL-mut+ miR-24-3p-mimic groups. 293T cells were harvested 30 h after transfection, and the firefly luciferin signal and the Renilla fluorescein signal were measured using a fluorescence luminescence detector according to the dual luciferase reporter assay kit. Simultaneously, the study verified the miR-24-3p target gene NRP1 by dual luciferase reporter assay according to the above procedure.

### RNA-binding protein immunoprecipitation (RIP) assay

Based on the instruction manual, the RIP Kit (Millipore, Bedford, MA, USA) was used for the RIP assay. First, transfected cells were collected and lysed using RIP lysis buffer. Next, RIP buffer containing magnetic beads conjugated with human anti-Ago2 antibody was added to the cell extract for incubation. Then, proteinase K was applied to digest the protein and immunoprecipitated RNA was separated. RT-qPCR was performed to detect THRIL and miR-24-3p.

### Statistical analysis

SPSS 17.0 (SPSS, Inc., Chicago, IL, USA) was used for data analysis. The experimental data are expressed as mean ± standard deviation (SD). For data comparisons in multiple groups, one-way ANOVA with Turkey’s post hoc test was performed. A two-tailed paired t-test was used to compare differences between two groups being compared once. Differences were considered statistically significant at P < 0.05.

### Availability of data and materials

All data from this study are available on a reasonable request in this published article.

## References

[1] Ros HH, Booij LH, de Lange JJ. The use of neurophysiologic monitoring during anaesthesia. Int J Clin Monit Comput. 1987; 4:179–84. 10.1007/BF02915905 3655509

[2] Allahtavakoli M, Shabanzadeh A, Roohbakhsh A, Pourshanazari A. Combination therapy of rosiglitazone, a peroxisome proliferator-activated receptor-gamma ligand, and NMDA receptor antagonist (MK-801) on experimental embolic stroke in rats. Basic Clin Pharmacol Toxicol. 2007; 101:309–14. 10.1111/j.1742-7843.2007.00127.x 17910613

[3] Hayashida K, Sano M, Ohsawa I, Shinmura K, Tamaki K, Kimura K, Endo J, Katayama T, Kawamura A, Kohsaka S, Makino S, Ohta S, Ogawa S, Fukuda K. Inhalation of hydrogen gas reduces infarct size in the rat model of myocardial ischemia-reperfusion injury. Biochem Biophys Res Commun. 2008; 373:30–35. 10.1016/j.bbrc.2008.05.165 18541148

[4] Wakida Y, Nordlander R, Kobayashi S, Kar S, Haendchen R, Corday E. Short-term synchronized retroperfusion before reperfusion reduces infarct size after prolonged ischemia in dogs. Circulation. 1993; 88:2370–80. 10.1161/01.cir.88.5.2370 8222130

[5] Losordo D. inhibition of src for treatment of reperfusion injury related to revascularization. 2006.

[6] Xu B, Jin X, Yang T, Zhang Y, Liu S, Wu L, Ying H, Wang Z. Upregulated lncRNA THRIL/TNF-α Signals Promote Cell Growth and Predict Poor Clinical Outcomes of Osteosarcoma. Onco Targets Ther. 2020; 13:119–29. 10.2147/OTT.S235798 32021260 PMC6954829

[7] Deng Y, Luan S, Zhang Q, Xiao Y. Long noncoding RNA THRIL contributes in lipopolysaccharide-induced HK-2 cells injury by sponging miR-34a. J Cell Biochem. 2018. [Epub ahead of print]. 10.1002/jcb.27354 30414207

[8] Xia J, Jiang N, Li Y, Wei Y, Zhang X. The long noncoding RNA THRIL knockdown protects hypoxia-induced injuries of H9C2 cells through regulating miR-99a. Cardiol J. 2019; 26:564–74. 10.5603/CJ.a2018.0054 29745968 PMC8084388

[9] Liu G, Wang Y, Zhang M, Zhang Q. Long non-coding RNA THRIL promotes LPS-induced inflammatory injury by down-regulating microRNA-125b in ATDC5 cells. Int Immunopharmacol. 2019; 66:354–61. 10.1016/j.intimp.2018.11.038 30521964

[10] Moharamoghli M, Hassan-Zadeh V, Dolatshahi E, Alizadeh Z, Farazmand A. The expression of GAS5, THRIL, and RMRP lncRNAs is increased in T cells of patients with rheumatoid arthritis. Clin Rheumatol. 2019; 38:3073–80. 10.1007/s10067-019-04694-z 31346885

[11] Liu T, Liu J, Tian C, Wang H, Wen M, Yan M. LncRNA THRIL is upregulated in sepsis and sponges miR-19a to upregulate TNF-α in human bronchial epithelial cells. J Inflamm (Lond). 2020; 17:31. 10.1186/s12950-020-00259-z 32944003 PMC7488348

[12] Maegdefessel L, Spin JM, Raaz U, Eken SM, Toh R, Azuma J, Adam M, Nakagami F, Heymann HM, Chernogubova E, Jin H, Roy J, Hultgren R, et al. Erratum: miR-24 limits aortic vascular inflammation and murine abdominal aneurysm development. Nat Commun. 2015; 6:6506. 10.1038/ncomms7506 25716653 PMC4349130

[13] Xiao X, Lu Z, Lin V, May A, Shaw DH, Wang Z, Che B, Tran K, Du H, Shaw PX. MicroRNA miR-24-3p reduces apoptosis and regulates Keap1-Nrf2 pathway in mouse cardiomyocytes responding to ischemia/reperfusion injury. Oxid Med Cell Longev. 2018; 2018:7042105. 10.1155/2018/7042105 30622671 PMC6304907

[14] Tan H, Qi J, Fan BY, Zhang J, Su FF, Wang HT. MicroRNA-24-3p attenuates myocardial ischemia/reperfusion injury by suppressing RIPK1 expression in mice. Cell Physiol Biochem. 2018; 51:46–62. 10.1159/000495161 30439713

[15] Wei W, Peng J, Shen T. Rosuvastatin alleviates ischemia/reperfusion injury in cardiomyocytes by downregulating Hsa-miR-24-3p to target upregulated uncoupling protein 2. Cell Reprogram. 2019; 21:99–107. 10.1089/cell.2018.0039 30835496

[16] Jiang SX, Sheldrick M, Desbois A, Slinn J, Hou ST. Neuropilin-1 is a direct target of the transcription factor E2F1 during cerebral ischemia-induced neuronal death *in vivo*. Mol Cell Biol. 2007; 27:1696–705. 10.1128/MCB.01760-06 17178835 PMC1820462

[17] Jiang SX, Whitehead S, Aylsworth A, Slinn J, Zurakowski B, Chan K, Li J, Hou ST. Neuropilin 1 directly interacts with Fer kinase to mediate semaphorin 3A-induced death of cortical neurons. J Biol Chem. 2010; 285:9908–18. 10.1074/jbc.M109.080689 20133938 PMC2843238

[18] Glinka Y, Mohammed N, Subramaniam V, Jothy S, Prud'homme GJ. Neuropilin-1 is expressed by breast cancer stem-like cells and is linked to NF-κB activation and tumor sphere formation. Biochem Biophys Res Commun. 2012; 425:775–80. 10.1016/j.bbrc.2012.07.151 22885184

[19] Dong L, Hou R, Xu Y, Yuan J, Li L, Zheng C, Zhao H. Analyzing the Correlation between the Level of Serum Markers and Ischemic Cerebral Vascular Disease by Multiple Parameters. Comput Math Methods Med. 2015; 2015:972851. 10.1155/2015/972851 26609318 PMC4644555

[20] Zhu M, Dai J, Li S. Cerebral angiography and MR perfusion images in patients with ischemic cerebral vascular disease. Chin Med J (Engl). 2002; 115:1687–91. 12609089

[21] Sayad A, Hajifathali A, Omrani MD, Arsang-Jang S, Hamidieh AA, Taheri M. Expression of TNF- and HNRNPL-related immunoregulatory long non-coding RNA (THRIL) in acute myeloid leukemia: is there any correlation? Iran J Allergy Asthma Immunol. 2018; 17:274–80. 29908545

[22] Eftekharian MM, Ghafouri-Fard S, Soudyab M, Omrani MD, Rahimi M, Sayad A, Komaki A, Mazdeh M, Taheri M. Expression analysis of long non-coding RNAs in the blood of multiple sclerosis patients. J Mol Neurosci. 2017; 63:333–41. 10.1007/s12031-017-0982-1 28967047

[23] Li Z, Chao TC, Chang KY, Lin N, Patil VS, Shimizu C, Head SR, Burns JC, Rana TM. The long noncoding RNA THRIL regulates TNFα expression through its interaction with hnRNPL. Proc Natl Acad Sci USA. 2014; 111:1002–07. 10.1073/pnas.1313768111 24371310 PMC3903238

[24] Evangelista V, Dell'Elba G, Celardo A, Manarini S, Cerletti C. TxA2-mediated myocardial ischemia as a consequence of an acute lung inflammatory reaction in the rabbit. J Thromb Haemost. 2003; 1:314–9. 10.1046/j.1538-7836.2003.00067.x 12871506

[25] Kaji M, Inoue K, Kinoshita H. [Effects of long-term preoperative administration of low-dose erythromycin on warm ischemia-reperfusion pulmonary injury]. Nihon Kokyuki Gakkai Zasshi. 1998; 36:939–47. 9916477

[26] Thiex R, Tsirka SE. Brain edema after intracerebral hemorrhage: mechanisms, treatment options, management strategies, and operative indications. Neurosurg Focus. 2007; 22:E6. 10.3171/foc.2007.22.5.7 17613237

[27] Bułdak Ł, Machnik G, Bułdak RJ, Łabuzek K, Bołdys A, Belowski D, Basiak M, Okopień B. Exenatide (a GLP-1 agonist) expresses anti-inflammatory properties in cultured human monocytes/macrophages in a protein kinase A and B/Akt manner. Pharmacol Rep. 2016; 68:329–37. 10.1016/j.pharep.2015.10.008 26922535

[28] Sheng C, Hu F, Wu L. Geniposide alleviates hypoxia-induced injury by down-regulation of lncRNA THRIL in rat cardiomyocytes derived H9c2 cells. Eur J Pharmacol. 2019; 854:28–38. 10.1016/j.ejphar.2019.03.058 30953616

[29] Xu W, Liu M, Peng X, Zhou P, Zhou J, Xu K, Xu H, Jiang S. miR-24-3p and miR-27a-3p promote cell proliferation in glioma cells via cooperative regulation of MXI1. Int J Oncol. 2013; 42:757–66. 10.3892/ijo.2012.1742 23254855

[30] Zhu L. The dynamic changes of neurons and astrocytes and their cyclin d1 expression difference in rats after cerebral ischemia/reperfusion injury. Chinese Journal of Histochemistry & Cytochemistry. 2008.

[31] Whitehead SN, Gangaraju S, Slinn J, Hou ST. Transient and bilateral increase in neuropilin-1, Fer kinase and collapsin response mediator proteins within membrane rafts following unilateral occlusion of the middle cerebral artery in mouse. Brain Res. 2010; 1344:209–16. 10.1016/j.brainres.2010.05.036 20493826

[32] Luo Y, Raible D, Raper JA. Collapsin: a protein in brain that induces the collapse and paralysis of neuronal growth cones. Cell. 1993; 75:217–27. 10.1016/0092-8674(93)80064-l 8402908

[33] Fujita H, Zhang B, Sato K, Tanaka J, Sakanaka M. Expressions of neuropilin-1, neuropilin-2 and semaphorin 3A mRNA in the rat brain after middle cerebral artery occlusion. Brain Res. 2001; 914:1–14. 10.1016/s0006-8993(01)02765-2 11578592

[34] Shirvan A, Ziv I, Fleminger G, Shina R, He Z, Brudo I, Melamed E, Barzilai A. Semaphorins as mediators of neuronal apoptosis. J Neurochem. 1999; 73:961–71. 10.1046/j.1471-4159.1999.0730961.x 10461885

[35] Oosthuyse B, Moons L, Storkebaum E, Beck H, Nuyens D, Brusselmans K, Van Dorpe J, Hellings P, Gorselink M, Heymans S, Theilmeier G, Dewerchin M, Laudenbach V, et al. Deletion of the hypoxia-response element in the vascular endothelial growth factor promoter causes motor neuron degeneration. Nat Genet. 2001; 28:131–38. 10.1038/88842 11381259

[36] Shi CX, Ding YB, Jin FY, Li T, Ma JH, Qiao LY, Pan WZ, Li KZ. Effects of sevoflurane post-conditioning in cerebral ischemia-reperfusion injury via TLR4/NF-κB pathway in rats. Eur Rev Med Pharmacol Sci. 2018; 22:1770–75. 10.26355/eurrev_201803_14595 29630125

[37] Xue X, Qu XJ, Yang Y, Sheng XH, Cheng F, Jiang EN, Wang JH, Bu W, Liu ZP. Baicalin attenuates focal cerebral ischemic reperfusion injury through inhibition of nuclear factor κB p65 activation. Biochem Biophys Res Commun. 2010; 403:398–404. 10.1016/j.bbrc.2010.11.042 21093411

